# Correction: Smartphone-read phage lateral flow assay for point-of-care detection of infection

**DOI:** 10.1039/d4an90016b

**Published:** 2024-02-13

**Authors:** Maede Chabi, Binh Vu, Kristen Brosamer, Maxwell Smith, Dimple Chavan, Jacinta C. Conrad, Richard C. Willson, Katerina Kourentzi

**Affiliations:** a Department of Biomedical Engineering, University of Houston Houston Texas 77204 USA willson@uh.edu; b Department of Chemical and Biomolecular Engineering, University of Houston Houston Texas 77204 USA jcconrad@uh.edu edkourentzi@uh.edu; c Department of Biology and Biochemistry, University of Houston Houston Texas 77204 USA; d Escuela de Medicina y Ciencias de Salud, Tecnológico de Monterrey Monterrey Nuevo León 64710 Mexico

## Abstract

Correction for ‘Smartphone-read phage lateral flow assay for point-of-care detection of infection’ by Maede Chabi, *et al.*, *Analyst*, 2023, **148**, 839–848, https://doi.org/10.1039/D2AN01499H.

The authors regret that a complete Conflicts of interest section was not shown in the original article. The correct Conflicts of interest section is shown below.

## Conflicts of interest

Authors Vu and Willson are named inventors on IP which could relate to the subject of this paper. Willson, Kourentzi, and Vu acknowledge their financial interest in Glow Nanotech which at the time was developing a different LFA technology.

The Royal Society of Chemistry apologises for these errors and any consequent inconvenience to authors and readers.

## Supplementary Material

